# Living less safely through the pandemic in England for people with serious mental and physical health conditions: qualitative interviews with service users and carers of Black African, Caribbean, and South-Asian descent

**DOI:** 10.1186/s12889-024-20107-6

**Published:** 2024-10-05

**Authors:** Josephine Ocloo, Ruth Stuart, Hannah K. Dasch, Jacqui Dyer, Dina Choudhury, Leroy McAnuff, Stephen McGowan, Ioannis Bakolis, Jayati Das-Munshi

**Affiliations:** 1 Centre for Implementation Science, Department of Health Service and Population Research, Institute for Psychiatry, Psychology and Neuroscience, King’s College London, De Crespigny Park, London, UK; 2 National Institute for Health Research (NIHR) Applied Research Collaboration (ARC) South London, Institute of Psychiatry, Psychology & Neuroscience, King’s College London, De Crespigny Park, London, UK; 3 Department of Psychological Medicine, Institute of Psychiatry, Psychology and Neuroscience, King’s College London, De Crespigny Park, London, UK; 4 Department of Psychological Medicine, King’s College London, Weston Education Centre, Cutcombe Road, London, SE5 9RJ UK; 5 Black Thrive Global, Great Portland Street, London, UK; 6 National Institute for Health Research (NIHR) Maudsley Biomedical Research Centre (BRC), Hosted By South London and Maudsley NHS Foundation Trust and King’s College London, Denmark Hill, London, UK; 7 South London and Maudsley NHS Foundation Trust, Maudsley Hospital, Denmark Hill, London, UK; 8 ESRC Centre for Society and Mental Health, King’s College London, Aldwych, London, UK

**Keywords:** Ethnic Inequalities, Mental Health, Covid-19 Pandemic, Racism, Intersectional Discrimination, Peer Research

## Abstract

**Background:**

COVID-19 Ethnic Inequalities in Mental health and Multimorbidities (COVEIMM) is a mixed methods study to explore whether COVID-19 exacerbated ethnic health inequalities in adults with serious mental and physical health conditions. We analysed data from electronic health records for England and conducted interviews in Birmingham and Solihull, Manchester, and South London. Sites were selected because they were pilot sites for the Patient and Carer Race Equality Framework being introduced by NHS England to tackle race inequalities in mental health. Prior to the pandemic people in England with severe mental illnesses (SMIs) faced an 11–17-year reduction in life expectancy, mostly due to preventable, long-term, physical health conditions. During the pandemic there was a marked increase in deaths of those living with an SMI.

**Aims:**

This qualitative interview study aimed to understand the reasons underlying ethnic inequalities in mortality and service use during the COVID-19 pandemic for adult service users and carers of Black African, Black Caribbean, Indian, Pakistani, and Bangladeshi backgrounds living with serious multiple long-term mental and physical health conditions.

**Methods:**

We took a participatory action research approach and qualitative interviews undertaken by experts-by-experience and university researchers Participants were purposively sampled by ethnicity, diagnoses, and comorbidities across three geographically distinct sites in England. Transcriptions were coded inductively and deductively and analysed thematically.

**Results:**

Findings indicated multiple points along primary and secondary health pathways for mental and physical health that have the potential to exacerbate the unjust gap in mortality that exists for Black and Asian people with SMIs. Issues such as timely access to care (face-to-face and remote), being treated in a culturally appropriate manner with empathy, dignity and respect, and being able to use services without experiencing undue force, racism or other forms of intersectional discrimination were important themes arising from interviews.

**Conclusion:**

These poor experiences create systemic and enduring healthcare harms for racialised groups with SMIs that need to be addressed. Our findings suggest a need to address these, not only in mental health providers, but across the whole health and care system and a need to ensure more equitable healthcare partnerships with service users, carers, and communities from racialised backgrounds who are often excluded.

**Supplementary Information:**

The online version contains supplementary material available at 10.1186/s12889-024-20107-6.

## Background

Research has shown that ethnic minority individuals have long reported poorer healthcare experiences when compared to White individuals [1]. The COVID-19 pandemic has magnified healthcare inequalities in the UK for minority ethnic groups [2]. Black and Asian patients were shown to have required hospitalisation with COVID-19 during the pandemic more frequently than their White counterparts, with poorer outcomes and a greater chance of mortality [3, 4]. Pre-existing medical conditions were factors in increasing the risk of death from COVID-19 [5] and links between comorbidity, social deprivation and ethnicity were evident in higher rates of illness and increased severity [6]. Populations from minority ethnic backgrounds are more likely to live in deprived areas [7] and to work in occupations which increased their exposure to infection during the pandemic [8]. Even accounting for deprivation and prior medical conditions, populations from Black and South Asian backgrounds still showed a significantly increased risk of dying from COVID-19 [5]. More generally, people from minority ethnic backgrounds experience stark inequalities within the UK mental healthcare system and are more likely to be diagnosed with a severe mental illness (SMI) [9]. Those living with an SMI, particularly mood disorders and schizophrenia, were shown to have had a marked increase in deaths during the COVID-19-pandemic [10–12].

Evidence shows that people with SMI experience an unjust health gap[13] constituting an 11–17-year reduction in life expectancy in comparison to the wider population [14] and have higher levels of common, preventable, physical health comorbidities [15]. The pandemic led to changes in both physical and mental health service provision with adaptations such as remote working significantly affecting the ability to access services [16]. Public health messages led to patients with SMI not seeking treatment for non-COVID-19 related physical health needs, due to concerns around impacting acute services at the height of the pandemic [17]. In areas with large ethnically diverse populations and elevated levels of deprivation, there was a noted decrease in non-COVID-19 planned and emergency admissions [18].

Much has already been written about the over-representation of minority ethnic patients within the mental health system. Black people are over four times more likely to be detained under the Mental Health Act 1983 (MHA)^1^ than White people and more than eleven times more likely to be subject to a Community Treatment Order (CTO) [19] under which patients can be recalled to hospital if they refuse treatment.

The draft Mental Health Bill to amend the Mental Health Act 1983 for England and Wales constituted much anticipated and long overdue reform to the Mental Health Act. The amendment was a key commitment in the Conservative Party manifestos of both 2017 and 2019. The draft bill contained a range of measures aimed at shifting the balance of power to place patients and service users at the centre of decisions on their care. It was also an opportunity to address the considerable racial disparities and injustices in the use of the Act with Black people in particular and other racialised groups [20]. Despite this, the UK government’s new legislative programme set out in the King’s speech on the 7th of November 2023 failed to include the Mental Health Bill. These reforms have once again recently been highlighted by the UK government as requiring reform [see King’s Speech 2024 Programme^2^] Our findings show that this reform is fundamental to address the considerable levels of racism, unfair and forced levels of treatment, rather than therapeutic and person-centred care that Black (in particular) and Asian people face with SMIs and in their physical healthcare (as discussed further below).

We conducted a mixed methods (quantitative and qualitative) study, with the qualitative findings reported in this paper. We aimed to use an innovative mixed methods design [21], working with large-scale electronic health records, representative of local ethnically diverse populations, as well as with nationally representative samples from the UK. Quantitative insights were further informed by qualitative research with data collection co-produced with people with lived experience of using mental health services, who also identify as being of a minority ethnic background as reported in this study.

We aimed to:

- understand the reasons underlying ethnic inequalities in mortality and service use during the COVID-19 pandemic for service users and carers of Black African, Black Caribbean, Indian, Pakistani, and Bangladeshi backgrounds living with multiple serious long-term mental and physical health conditions.
- To understand issues of access and barriers to care, and discrimination within health services for these groups.

Central to our approach was our collaboration with individuals involved in the Patient and Carer Race Equality Framework (PCREF) initiative working in the three pilot sites in the study. The PCREF is an anti-racism framework which is being implemented in England, through NHS England, and which seeks to directly tackle race inequalities in mental health [22]. Developing the PCREF was one of the main recommendations of an independent review of the MHA in 2018 [23]. This aimed to address discrimination, bias, and racial inequalities within mental health care through ensuring that mental health trusts are working according to statutory and legislative requirements (e.g., Equality Act 2010) as well as addressing the cultural and behavioural shifts needed to improve care [24].

A key aim of the PCREF is the requirement to co-produce and implement the framework in partnership with minority ethnic groups and communities. The requirement for patient and public involvement (PPI) has been long-standing in mental health, but mental health services have been criticised for failing to effectively engage Black, Asian and minority groups [25] and for pathologising the behaviour of these populations [26]. Given this context, the study used a participatory action research (PAR) approach. This is relevant to the development of co-produced research with patients, the public and communities as it seeks to challenge the control of how knowledge is defined, to create more equal partnerships as described in the methods below [27, 28].

## Methods

### Study design

PCREF national approaches were initially informed by findings at three ‘pilot’ sites (Manchester, Birmingham & Solihull, and Lambeth (in southeast London). Therefore sites for recruitment to our qualitative study were identified on this basis. Input from representatives from the PCREF pilot sites on the study Steering Group was particularly useful in feeding into the design of the study and interview questions and shaping the study in terms of its collaborative nature, which is a key aspect of the PCREF approach, with input from peer researchers. The study findings will in turn inform ongoing PCREF work in the pilot sites.

We drew upon a community peer research approach. This is a participatory method which enabled us to engage with the communities directly affected by the research and enable them to take part in designing, conducting, analysing, and disseminating the research. In the field of mental health, peer research models have long been used to enable those with lived experience of using mental health services to be involved in research[29]. The use of peer research approaches is particularly successful when conducted within a theoretical model like Participatory Action Research (PAR), which supports an ongoing commitment to collaboration, co-learning, mutual leadership, and shared decision-making[30]. We also drew upon the GRIPP2 short form in reporting the results of patient and public involvement in this study[31] and followed CORE-Q reporting guidelines for qualitative research[32].

We were advised by a Steering Group consisting of university researchers [6], peer researchers [4], a representative from Black Thrive Global [1] and three NHS mental health trusts [3] which were piloting the PCREF in England [Birmingham and Solihull, Manchester, and South London], at the time of this study. Black Thrive Global (a collaborator on the study, based in South London), is a community interest company that works collaboratively with communities, statutory organisations, voluntary groups, and the private sector to reduce mental health inequalities. Members of the Steering Group fed into all aspects of the study design and research process.

### Peer researcher involvement

The qualitative research was co-produced by a group of experts-by-experience and occupation: three university researchers and four peer researchers with lived experience of using mental health services. Peer researcher involvement was crucial to the study, with the aim to have someone involved from each of the three geographical regions. We asked our three pilot PCREF sites to circulate information on the peer researcher roles and payment. We received interest from and met various people and were able to select four people representing all the geographical regions. All the peer researchers were from minority ethnic backgrounds with personal experience of using mental health services. One had been involved with PCREF work, but didn’t have research experience, two others had been involved with research previously and one was a public member on a Trust committee who wanted to gain more experience with involvement in research. The peer researchers were involved in all aspects of the study, the initial design through discussions in the Steering Group, feeding into and reviewing and piloting the topic guides, helping with participant recruitment, conducting interviews and analysis, write-up for publication and involvement with an online dissemination event (discussed further below and in the sections on Participant Recruitment, Conducting Interviews and Analysis). Incentives for peer researcher participation included opportunities to engage with the work throughout all stages of the study and to be paid for this at a rate of £25 per hour, as well as participating in training sessions if they wished and being involved in wider dissemination of the findings.

Peer researchers DC, LMN and SJ were provided with qualitative research and interview training in an online meeting, after email communication with the peer researchers to see if they would find this useful. The session was conducted by JO who had considerable experience of working with public members as an activist/researcher and with lived experience researchers in research projects. Opportunities were subsequently provided for the peer researchers to practise their interviewing skills and to pilot the topic guide with research assistant (HD). For the training session JO put together key slides, making sure that the information was not presented in an over-complex and over theoretical way which might overwhelm the peer researchers attending. Plenty of time was allowed for questions, clarification, reflection and discussion in the session and everyone was reimbursed for their time. Three peer researchers and university researcher [JO], interviewed twenty-four service users and six carers between 28 July—28 October 2021 (see Table 1) until it was felt by the team we had a good spread of interviews overall and data saturation was reached. Study researchers HKD and JO facilitated the peer researchers to conduct their interviews by being present online to manage the recording, address any technological problems, and to help respond to any participant concerns or distress, if needed.

**Table 1:** Participant role, location, ethnic background, gender, sexual orientation, age, and disability

**Participants interviewed:**	*N* = 32
^a^Carers, Service users and Voluntary Sector Professionals	32
**Place of residence:**	*N* = 32
^a^Birmingham & Solihull and Manchester	15
London	17
**Ethnic background:**	*N* = 32
African	10
Asian	10
Caribbean	12
**Gender:**	*N* = 32
female (including trans)	17
male (including trans)	15
same as at birth	29
**Sexual orientation (service users and carers):**	*N* = 30
^a^Gay and Lesbian	4
heterosexual	26
**Age (service users and carers):**	*N* = 30
^a^18-54	18
^a^55-74	12
**Disability (service users and carers):**	*N* = 30
none	15
^a^Mental and physical disabilities	15

^a^An asterisk has been used to indicate where smaller categories have been combined to avoid identifying participants

Key positive outcomes from collaborating with peer researchers enabled experiential knowledge to shape the whole design of the study, in particular helping to get the focus of the questions right, being able to recruit racialised groups meeting the study criteria and to help identify key themes, conclusions, and outcomes for action. Targeted researcher support for the peer researchers was crucial to enabling their involvement, although this presented challenges in terms of the rapid timescales of the study.

### Participant recruitment

Service user participants were purposively sampled by age (18 +), self-ascribed ethnicity (Black African, Black Caribbean, Bangladeshi, Indian, or Pakistani descent), self-reported mental health diagnoses and physical long-term health conditions or a history of previous contact, with secondary mental health services in England. (see Table 2 for details). The inclusion criteria for carers were based upon them being aged 18 + and caring for someone who met the service user criteria above. Some carers also reported mental and physical health conditions.

**Table 2:** Service user and carers’ self-reported physical health conditions and mental health diagnoses

Physical health conditions	*N* = 30	Mental health diagnoses	*N* = 30
Arthritis	2	Anorexia nervosa	1
Asthma	5	Anxiety/acute anxiety	2
Autoimmune diseases	1	Bipolar disorder	9
Cancer	1	Depression/acute, chronic, major, or severe depression	13
Chronic fatigue	1	Obsessive Compulsive Disorder (OCD)	1
Chronic pain	2	Personality disorder or Emotionally Unstable Personality Disorder (EUPD)	2
Diabetes or pre-diabetes	12	Psychosis/schizophrenia/hallucinations	7
Disability affecting gait	1	Post Traumatic Stress Disorder (PTSD) or complex PTSD	5
Eye condition	1		
Fibromyalgia	1		
Head/spinal cord injury	1		
Heart condition/disease	2		
High cholesterol	1		
HIV	2		
Hypertension	3		
Sarcoidosis	1		
Slipped discs	1		
Stomach/diaphragm issues	1		
Tinnitus	1		
Underactive thyroid	1		

We used multiple recruitment methods for service users and carers. Steering Group members were asked to circulate specific flyers aimed at these groups, and Global Black Thrive and the pilot mental health trusts were asked to post information about the study on their websites. We used snowballing approaches to identify relevant groups and emailed community organisations in the geographical areas of the pilot sites such as African and Caribbean Mental Health Services (ACMHS) in Manchester, and posted on social media channels to further recruit from local catchment areas. One peer researcher in the Steering Group (DC) was especially active and effective, identifying, telephoning, and personally visiting more than twenty local community organisations in Birmingham and Solihull to ask if they knew of people who met the narrow inclusion criteria for service users and carers and who might be willing to be interviewed. This increased the sample size in this area when, few responses were coming through other routes. Mosaic Clubhouse based in South London (a non-profit organisation that provides support and opportunities for people with mental health conditions) also proved to be crucial in helping recruit participants through their networks, particularly those people who needed help with joining meetings online (as described in the next section). Payment incentives were £20 per interview, but more broadly many participants were keen to have an opportunity to articulate their experiences as Black and Asian people as part of a research study that would eventually be published, and which might have an impact in changing policy and practice in mental health.

### Conducting interviews

HKD arranged the interviews by email and telephone, and they were conducted online via Microsoft Teams and Zoom, or on a freephone telephone number. Several participants, who did not feel able to use the technology on their own, needed assistance. Mosaic Clubhouse facilitated some interviews by providing computers and an internet connection in a private room. Study information sheets and consent forms for service users and carers were emailed to all potential participants in advance of the interviews. Consent was recorded separately on Teams or Zoom immediately prior to the interviews.

The semi-structured interviews ranged in length from 30–98 min (mean length 60 min). Different topic guides were used for service users and carers, but both sought to understand the reasons underlying ethnic inequalities in mortality and service use during the COVID-19 pandemic and to understand access and barriers to care, and discrimination within health services. The topic guides contained prompts and statements to support the peer researchers to conduct the interviews. Several participants expressed positive comments and seemed reassured by being told that peer researchers were conducting their interviews.

Demographic questions were asked at the end of the interviews when participants were more at ease and recording had stopped. All questions were optional: participants could choose ‘prefer not to say’. JO or HKD noted on a spreadsheet each participant’s age category, ethnic background, gender, sex at birth, sexual orientation, and whether they had a disability or impairment (see Table 1).

In November 2021, JO also interviewed representatives from two community groups for their perspectives: the Programme Manager at Mosaic Clubhouse, and the Advice Services Co-ordinator at Ashiana Community Project (ACP). ACP provides multilingual support to improve the quality of life for people in the Sparkbrook area of Birmingham. Their services are not exclusively for people with mental health conditions but many of their clients do have mental health challenges.

### Analysis

All interviews were transcribed verbatim by a professional ethically approved GDPR compliant transcription service, which was a requirement of our ethics approval. RS and HKD checked the transcripts for accuracy and pseudonymised them. A framework approach was used for the analysis to provide a structured and focussed way of looking at the large number of qualitative interviews thematically in an in-depth way, whilst staying focussed on the research questions. Peer researchers (DC, SJ, YT) read five different transcripts and met with JO, RS, HKD to discuss potential themes which fed into the coding framework. JO then coded all the transcripts in NVivo 12 using a coding analysis frame of inductive themes identified at the meeting and deductive codes based on questions in the topic guides. RS and HKD assisted with analysis of some sections of the coded data and produced theme summaries.

As part of the participatory approach, the study team (JO, RS, HKD, DC, LMN, SJ) wrote individual reflective logs to capture their experiences of the research process. University researchers self-identified as White females [2] and Black female [1]. In terms of our collective standpoints with this work, there was a strong recognition of the need to address imbalances of power to enable peer researcher to be involved through providing extra ongoing support for them in the research process. For example, getting in touch by telephone and posting paper documents rather than just using email, creating opportunities to contribute verbally via individual online meetings rather than in writing, and affording sufficient time for preparation and debriefing. The involvement of the university researcher from a racialised background, was seen as a particular strength within the research team in enabling open discussions about emerging themes of racism in the interview data and more generally in the research. Peer researchers fed back about their experiences of recruiting and interviewing, for example, recommending that the language be simplified in the consent statements which they had to read out to participants. Challenges for the university team included being able to provide extra time to allow the peer researchers to be involved, whilst getting the balance right in terms of communicating with peer researchers about work that needed to be progressed and completed to tight deadlines.

After analysis of the data, participants were invited to an online meeting co-facilitated with our peer researchers to discuss the findings and make recommendations (Table 4 Appendix 1). Peer researchers verbally commented on the manuscript, with the support of a university researcher [SMG], who wrote up their comments and incorporated them into the paper.

## Results

### Managing mental health and wellbeing during the pandemic

Thirty-two semi-structured qualitative interviews were conducted online with service users and carers of Black African, Black Caribbean, Indian, Pakistani, or Bangladeshi descent (Table 1), and two community groups. Most of the sample resided in London, with remaining participants from either Birmingham or Manchester. All the respondents had multiple long-term serious mental and physical health conditions, with many service users [11] and carers [3] having more than one physical health condition. This was captured through self-reported diagnosis by people who had been in contact with secondary mental healthcare and considered to be severely affected by mental health conditions (Table 2).

Their experiences are set out below under the headings that were identified as key themes from the findings.

Participants are uniquely identified below as carers or service users (C or SU) with a personal number, and by their location in one of the three pilot sites, for example ‘SU5-site3’.

More than two thirds of participants described being negatively impacted with their mental health during the pandemic. Many service users experienced a very serious deterioration, and others said that their mental health condition was impacted by depression.

*‘I would say it was the majority of the COVID period. I felt depressed and stressed. I would take a lot of overdoses and end up in hospital. Then later on, I started doing worse things, like trying to kill myself, jumping off a carpark or setting myself on fire or trying to get hit by a car, things like that’* [SU2-site2].

*‘I’ve diabetes, very high blood sugar. They told me it was off the scale, but nobody’d given me any advice about what to do. Then all these other illnesses have kicked off, and I can’t distinguish one illness from the other, and they affect my sleep, my mental health, terrible gut problems, digestive problems, severe constipation, and pains in me stomach and in most of my body, and chronic migraines. I had teeth coming out, so I was finding it hard to eat. Just feeling absolutely terrible every day, you just want to die’* [SU1-site2].

The majority of carers also struggled with their mental health, whilst managing a serious deterioration in the mental health of the family member they were caring for.

*‘I started to have hallucinations. I was hearing voices. My sleeping habits had been very bad. I was getting severe heartburns, indigestions. […] My mental wellbeing is getting better, but I would say the lockdown has dramatically made me very unwell’* [C1-site1].

Not everyone was negatively affected by the lockdown period. Some service users showed strong resilience and were able to change their behaviour positively: ‘*I’ve been able to adapt and love staying indoors and doing my stuff around my own house’* [SU4-site2]. Another noted he coped better because he was used to not having people around him so he ‘*already had some introduction into this way of living, way of life’* [SU5-site3].

### Impact of the pandemic on mental health and care services

More than two thirds of the service user participants said they felt their care had been impacted by the pandemic in different ways, leaving some struggling with little or no support, whilst others experienced much needed health and care services being seriously disrupted or unavailable.

Carers described losing the support they heavily relied on with support groups, voluntary and community organisations, day centres, social care, social services, and paid carer support either no longer available or accessible or replaced with a telephone or video call. Even when paid support was available, one carer, whose daughter received professional care four times a day, brought her daughter home to protect her from the risk of COVID-19 infection from multiple carers.

Another carer, with her own considerable health problems, described how affected she was by her husband’s deteriorating mental health in lockdown and trying to access care across different systems.

*‘He’s got bipolar. He’s diabetic. He’s had two stents in his heart. He’s a heavy smoker, especially when he’s a bit high [bi-polar disorder]. And he’s not sleeping at all. When I say high, he’s been very out of control, abusive, verbally, and mentally and everything. He was very challenging and hard to cope with’* [C3-site1].

When her husband was violent, she said she had to phone the police, because she was not able to get any mental health support during the pandemic, as she had done previously from the Home Treatment Team.

*‘The police arrested him, took him to the hospital and then they sent him home early in the morning. Normally the hospital have to admit him which didn’t happen. And I thought, where’s the help then? If they’re sending him back home after a situation like that, where’s the understanding? How do you explain yourself? That you really need the help before it’s too late?’* [C3-site1].

At the time of our interview, the carer and her husband had moved in with their son’s family, so that the son could intervene if the husband became violent.

Service users and carers described the detrimental impact of not being able to access mental health and hospital appointments or tests, and experiencing long waits on the telephone to get their appointments. Issues raised related to longer periods between appointments, shorter sessions, and poorer quality support online. These barriers caused major issues for participants in managing their various medical conditions across a complex health and care landscape.

The husband of the carer described above [C3-site1], said he was left feeling suicidal because he had got no mental health support:

‘*No, nothing. They ain’t bothered. If you’re dead, you’re dead. That’s it’* [SU3-site1].

A service user wondered if she had been left with no mental health support because of racism.

*‘We’ve only talked over the phone. Maybe it comes across to him as if I don’t need his help. I’ve been waiting for a coordinator for seven or eight months now, and that could be due to the COVID-19 as well, or it could be that they found out that I am a Black person that they think doesn’t need their help’* [SU5-site2].

Other participants described their distress at having to deal with endless barriers across the healthcare system in trying to access services and hospital appointments. One participant described becoming severely depressed with trying to sort out her missed hospital appointments, with no access to mental health care or support and she felt her GP [General Practitioner] had no time to support her with her severe depression. She was not able to use a computer or access Zoom at home.

*‘I have been traumatised by the experiences of the NHS. As far as I am concerned […] they have ruined our lives. […] In terms of mental health, I have been very suicidal, and I have been very unwell. I did not have the help and support that I needed, and the same with my mother and siblings as well. […] I have had to send a lot of complaints in because they have messed my appointments, they have cancelled them, rescheduled them, not communicated.’* [SU13-site3].

Another participant described the endless difficulties in the pandemic of managing multiple physical health conditions due to having an autoimmune disease and, asthma and diabetes, in addition to their mental health conditions. They talked about being denied access to mental health support after being ‘labelled’ with a personality disorder, and trying to manage their hospital appointments where they felt the quality of care had really deteriorated during the pandemic.

*‘I had a lot of appointments cancelled from the hospital and then I found that a lot of them have off rolled me. I’ve been discharged without even being told. And then a lot of the appointments, even now, they’re done on the telephone, and you find that, ‘cos they can’t see you, you can’t even point out things to them, like, ‘Look at my mouth,’ ‘Look at this.’ There’s no examination. And you find that the calls are really low, very poor consultation in terms of the person who’s ringing you up. Sometimes they’re not even the consultant; they’re some sort of student, and they’re having to say they don’t know what they’re doing, they don’t know why they’ve been asked to ring you, and they have no suggestion’* [SU1-site2].

They talked about being asked to travel miles two days before appointments to have a test for COVID-19 which incurred *‘massive costs in taxis’.* They could not travel by public transport because of their disabilities.

Issues were also raised about social care and being treated with compassion and dignity. A service user described her distress at not being able to get social care support to install shower facilities that she needed after having a bad fall due to her immune condition affecting her mobility. This condition had already badly affected her mental health and was made worse in the pandemic by not being able to get her various hospital appointments and help from social care. She feared long waiting lists would force her into the indignity of a situation where she might have to rely on her son to help her shower.

*‘At the moment, just walking in my flat I can fall or I can fall in the shower. But I find it humiliating, that I have to justify to social services and give an example like I might end up having to ask my son to help me in the shower’* [SU6-site3].

### Do health professionals know how to support you?

Interviewees were specifically asked whether health care professionals had understood how to work with them during the pandemic. A key theme was being able to have face-to-face contact in their care, and the importance of having access to their GPs. Half of the participants indicated they had a good relationship with their GP and would go and see them first about any health concerns.

*‘It would have to be my GP because she understands me, and she knows my condition…It’s made me feel tons better. When she talks to me with understanding and she helps me to get out of me bed, she helps me to start the day off positive’* [SU5-site2].

This GP was able to intervene quickly by writing to the patient’s psychiatrist when a problem emerged about the need for a change of tablets, which made a big difference to their mental health. The service user said: *‘That’s the only time I’ve had intervention without it being a crisis, and that’s because my GP wrote to the psychiatrist’* [SU5-site2]. This service user didn’t feel so positive about their relationship with their mental health officer [described earlier under the section ‘Impact of the Pandemic’], who she felt might not be providing her with support because of her ethnicity.

The carer described earlier who was left with no mental health support during the pandemic, despite her husband’s deteriorating mental health and violence towards her, said of her relationship with her GP: *‘My doctor … at the moment, he’s the only one that I can turn to, really’* [C3-site1].

Other respondents struggled to be heard, understood, and supported by their GPs. A participant who had a mental health crisis during the pandemic and spent two months in hospital described struggling to get a referral for a rheumatology appointment to address pain in her legs, feet and with symptoms to do with the menopause.

*‘I think sometimes GPs don’t understand what you’re going through. They block you from receiving help, because they’re not going through menopause; they don’t understand what you’re going through’* [SU14-site3].

Other participants reported being unable to get a GP appointment:

*‘It was horrible. I mean, deep into depression. It was terrible. As I said, October, November, December, where do you go? Who do you talk to? It was awful […] They didn’t want to talk to you. I wanted somebody face-to-face, but no. They’re still not seeing patients because of the COVID’* [SU3-site3]*.*

‘*it’s totally inaccessible trying to get a GP appointment, you used to be able to book online – they’ve stopped all that and they are not doing a lot of different services that they were doing before, which have been suspended because of the pandemic, but they’re carrying this on. Trying to make an appointment now at your doctors’, you’d be triaged by a receptionist – it’s very embarrassing, especially if you were there in person’* [SU1-site2].

Broader themes to emerge, when respondents described their contact with health care professionals, were about being able to have good relationships built on trust, being treated with compassion, dignity and respect, being listened to and not being discriminated against. The quote below, shows that despite the pandemic, individuals strove to provide the continuity in care that was otherwise so disrupted.

‘*My last CPN [Community Psychiatric Nurse], I had a good relationship with her, and she gradually got me back round to see my GP again’* [SU7-site3].

When relationships were not based upon respect, service users described situations impacting their privacy and dignity, such as being treated rudely, aggressively and with contempt, and being victimised when complaining.

A service user talked about taking their dying father to hospital during the pandemic and said, *‘there was this big, White British, bulky security guard and they were really rude and aggressive to us’* [SU13-site3]. They thought his behaviour in trying to supervise them was different to other families and based upon the fact they were Bangladeshi Muslim.

Another service user described being made *‘further unwell trying to sort out the chaos’* of their appointments, by healthcare staff who were: ‘*overly assertive, authoritative, officious’*. They said their mental and physical health conditions were now very bad and after being referred to an emergency NHS Access in Crisis service they said:

‘I *was really unwell waiting for this call. Then the call never came. And when it finally came, I told them how bad things are. I really needed to dig deep into the most traumatic, upsetting things that have happened to me, and they were just totally dismissive and saying to me, ‘Well, why are you not dead, then, if you say you’re suicidal?’ I felt I was being goaded’* [SU1-site2].

They felt some of their poor treatment was because they had ‘*complained about not being able to access services, and it’s retribution for that’*.

A service user who was acutely depressed described feeling *‘awful’* when left having to describe their symptoms outside in public to a receptionist when trying to see their GP:

‘They talk to you from the door outside. You can’t go in. That’s not just for me. You don’t want other people listening to you’ [SU3-site3].

These types of poor experiences and the feeling of being treated differently, directly impacted on the trust these individuals had in their relationships with healthcare professionals and services. Mosaic Clubhouse noted that lack of trust is very embedded in the Black community because of *“previous negative experiences with the mental health system……which leads to further inequalities in health care”.*

### Patient safety

Study participants were asked whether they had ever received unsafe care for their mental or physical health during the pandemic. Half of the participants described experiencing safety issues in their care. Ashiana community organisation expressed strong concerns about patient safety affecting people who used their organisation in the pandemic. Several different examples were given about what people thought was unsafe care and practice.

The site 1 carer [C3-site1] mentioned earlier, described how unsafe she felt when her husband became violent towards her when his mental health deteriorated, and she was not able to get any assistance from the police or mental health services. Other carers were concerned about the safety of the individuals they were caring for if they became ill during the pandemic or because no-one was checking up on them or providing support:

*‘If I ended up in hospital with COVID, there would have been a lot of issues or unsafe care in the sense that nobody would be looking out for her wellbeing’ or ‘behind doors she could have been receiving very bad care from me and nobody would have known any different’* [C1-site1].

A carer from site 3 was concerned about the safety of their daughter when attending medical appointments. The daughter had mental and physical disabilities following a car accident and the carer felt their daughter was not being given enough time to understand and respond to questions. They described what they said to their daughter to help her keep safe:

*‘You should write it down and try to plan. Just put down these things which we learnt to say how you can overcome and help yourself mentally and remember. So, I think it’s skills. When somebody’s asking, they don’t think that you may be slow and need a bit of time’* [C2-site3].

Service users also described safety issues to do with poor hospital care and treatment and not feeling supported:

*‘There is a pattern with the same hospital where people are just constantly dying, or they are being medically negligent in their care. It is even more so with ethnic minorities, like us, where there is institutional hierarchy and racism, and they just get away with it. They just think they are f…ing’ better than us when they are not. I am just so* angry’ [SU13-site3].

They said they would *‘rather have chest pains and die than go to A&E. And that is how we ethnic minorities feel, because we are so let down by the NHS services and by the authorities’* [SU13-site3].

Other respondents saw unsafe care to do with a lack of support and follow-up. The service user [described earlier], who had been waiting for a coordinator for seven or eight months, feared their health *‘could just deteriorate at any time. My mental health could just be just waiting to erupt and then it would be a crisis. I could have a fall, God forbid, and then where would I be? Back to square one’* [SU5-site2].

A different service user [SU2-site2] explained that they felt their care had suffered because of missed appointments and having online care where they could not be physically assessed properly for their mobility and joint pain. They had not been contacted by their mental health team for 18 months for their regular three-monthly appointments for blood tests and ECGs. They said these were essential appointments to monitor them due to the type of prescribed antipsychotic medication they were on. Their GP was crucial in getting the issue with the tests resolved.

Two further examples of unsafe care were described by men of African and Asian backgrounds [described later in ‘Being treated unfairly when trying to get help’], in relation to how they were treated by the police.

A service user from an African background felt he was unsafe because of being stereotyped and targeted adversely as a Black man, which he felt was even worse in the pandemic. He thought he was seen as more of a threat during the COVID-19 period. He was stopped by the police when walking on the streets and did not think it was because of his mental health condition:

‘*how does somebody know you have a mental health problem? You do not have a sign on you saying it’* [SU4-site3].

He felt his section was an *‘abuse of the Mental Health Act’:*

*‘*I *do not think that it was necessary, no’……. ‘And I am very sure, if I was White, it would not have happened’* [SU4-site3].

He said he also felt unsafe in his local mental health hospital because of what he considered to be racism in mental health care, the harmful effects of unnecessary physical restraint and the over prescription of his medication:

*‘I can tell you sodium valproate at 3000mg, combined with aripiprazole, combined with the ARTs that I take, the ARTs bump up the sodium valproate so that it is even above that’* [SU4-site3].

A service user from an Asian background said the main way they had felt unsafe was their treatment by the police when their mental health deteriorated previously before the pandemic. They described being held in a police station:

*‘I don’t see why they think that a police cell is better than going to hospital’… ’that’s not the place to be. You should be under care, but they’re saying they’ve got no space, so they put me in a cell’… ’even though they take the things out – your shoes, your laces, and things like that – I don’t know if you’ve heard of this word called ‘ligature points. But, to tell the truth, I know that probably there’s been deaths in custody, but they’ve not reported this, because them things, mental health is not news again’* [SU6-site2].

The community representative who worked with Ashiana thought that there were patient safety concerns throughout the pandemic with people dying because they couldn’t get the care they needed. This related to issues with GPs not making referrals, or people not getting appointments and/or not being taken seriously. In addition, concerns were raised about patient safety and neglect in hospital:

*“People were unsafe because no one cared for them, about them, health care was very unsafe during that time…“It’s definite. I know people that worked in the hospitals. They were telling us all sorts, like they haven’t got enough PPEs, they haven’t got the right procedures in place, they haven’t got anything, they’re stretched. And people have been left and neglected. Within the industry they were telling me this, within the hospital they were telling me this*.”

### Support from family, friends, and carers

These difficult health and care experiences were made worse for some people because many of them had no support from family or friends prior to the pandemic:

*“‘I don’t have any friends […] there’s been zero support for me […] probably that’s why I’ve got into such a terrible mess”* [SU1-site2].

Some service users described the absence of support as their own choice: to protect themselves from being re-traumatised by their family and to protect their neighbours from their anger; or because they are “*a germaphobe*”; or because “*it’s too depressing and too personal”* to ask friends for help. Others had no choice: they had no family from a young age and were brought up in ‘care’; or they had been shunned by close relatives, or the family members with whom they lived were unwell and in need themselves.

Service users who had support from friends but not family gave similar reasons for the lack of family support. They were ‘*estranged’*, in dispute, or their family was not in the UK; or one participant was tragically bereaved.

The pandemic and lockdowns were difficult for these participants, showing how the effects of COVID-19 had much wider social implications affecting health:

“*I just could not bear to deal with all the issues surrounding COVID, so I left my job […] I eventually stopped going out with certain friends […] I feel like I have not spoken to quite a few people in a while and I do not really know how to start those conversations up again. […] living on my own it was a lot more difficult to cope without going out and seeing people day to day […] I was just more depressed and distressed in terms of my mental health”* [SU2-site2].

Carer participants were all family members. A couple of carers, younger than 30, reported contact but no support from other family or friends despite the severity of the health conditions of the parents they were caring for (anorexia nervosa, cancer, psychosis, PTSD). Two other carers were themselves unwell. One with a heart condition and cognitive impairment, who was caring for their mother with diabetes, bipolar disorder, and depression, said:

*“…what really frustrates me is where friends and family will always say, ‘How’s your mum?’ and, if I say, ‘Mum’s well,’ they assume everything is perfect […] My mum can be well, but I can be in a terrible state …”* [C1-site1].

Another carer cited finances as a factor and reflected that they had been losing contact with family and friends prior to the pandemic.

*“my phone went down. It was the lack of finances because I am part time*” … *“It was limited before, but at least I knew that we had friends and family. They used to come every Saturday, and they just stopped coming. Our family was close, but all our meetings and all our get-togethers we don’t have anymore, it happened gradually and then COVID just actually cut it off completely. Even my neighbours have stopped talking. It’s very strange”* [C2-site3].

Conversely, in response to increased vulnerability due to the pandemic, several participants reported moving in with family, or friends moving in with them, whilst another service user was happy to have found a flat of their own. Many service users maintained social contact with friends by telephone and video during the pandemic but missed the visits and outings of pre-pandemic times. Some service users were helped through the pandemic by friends in a charitable fellowship and by church ‘*brothers and sisters’*.

### Support from community organisations

Community organisations were perceived to have played a particularly important role with our study participants during the pandemic. Participants received support from a range of places such as mental health charities and churches, and through adult education, exercise classes, and music therapy groups. Local charities for African, Asian, and Caribbean communities like the Ashiana Community Project in Birmingham, the African and Caribbean Mental Health Services organisation in Manchester, and the Caribbean and African Health Network were seen as especially important. About a third of our service user participants were supported by Mosaic Clubhouse in South London.

A service user who had not been a member of one of the participant community organisations before the pandemic, and who otherwise would have been completely isolated, was especially grateful for the weekly phone calls they received from a clubhouse engagement worker. Their GP had suggested this contact.

*“They are not therapists, they are not counsellors, but they were there to listen to me. They called me, every single day, to make sure I did not do something stupid to myself, to make sure that I had food in the house, because I stopped going out. They sent packages of food to me. They made sure I was eating well. They made sure that I was taking my medication. They made sure that I kept appointments at the hospital”* [SU9-site3].

One carer greatly appreciated that a local voluntary day centre which had closed, opened ‘*undercover’* during lockdowns for a limited number of people with mental illness:

“Even that one hour was helpful in a situation like this” [C3-site1].

However, some people received no support for either themselves or the relatives they were caring for.

### COVID-19 vaccination uptake

During the period of our interviews (27 July-2 November 2021) one or two doses of vaccine were offered to everyone aged 18 and older. Most of our participants were responsive to the vaccination programme and had already been double vaccinated. Some found it an easy decision to protect themselves, and some needed time to make up their minds. Others felt compelled to take up the vaccine fearing the new strains of the virus, or because of a previous experience of intensive care, or concerns due to shielding a partner**.**

Some participants had strong feelings about rejecting the vaccine after witnessing other people’s experiences.

*“…the vaccine is not 100% safe, and with what I’ve got already with me mental health and hearing the voices and what I’ve seen what’s happened to other people and how it’s affected my family, I don’t want to take it ... The only concern I’ve got is that I’m trying to keep well at the moment…”* [SU6-site2].

A close relative of another service user developed blood clots and died following vaccination. Another service user’s parent died from COVID-19, after treatment was initially denied and then delayed. At the time of the interview, the service user was too distressed and distrustful of health services to want to engage with them in any way.

A couple of unvaccinated carers expressed concerns about how the vaccine had been developed:

*“I think they need to be more tested… I think it’s a bit early for vaccines … I work in a hospital, and I’ve seen it with my colleagues …Quite a few of them got really ill, so that put me off”* [C4-site1].

### Receiving support by telephone or online

When asked about how they felt receiving their care by telephone or online during the pandemic, some service users described good experiences. Many participants expressed issues about receiving care in this way (see Table 3).

**Table 3:** Remote access issues reported by Service Users, Carers and Community Organisations

**Good Experiences**	**Wider Concerns**
Service Users/Carers	
• Care the same as before the pandemic, just online or by phone • Preference for digital/telehealth/video calls, which was helped by being given laptops/phones by family/voluntary sector organisations	• Preferring face to face contact as easier to see body language, build relationships, describe symptoms correctly • Difficult trying to communicate symptoms accurately ‘when mum speaks no English’ • Harder as a carer to convey abusive behaviour on the phone • Not liking digital meetings/concerns about digital security • Problems with access (or accessing online resources), because of poor internet connection • Brief poor-quality calls/assessments • Lacking computer equipment or skills, • Prefer to have email access to keep a record of contact • GP access difficult for appointments or medication, long waits on telephone if the person has little or no computer/IT skills
**Community Organisations [providing support]**	
• Mosaic Clubhouse gave all members laptops • Some members became ‘digital ambassadors’ to teach other members how to use the laptops • Staff helped members to make online bookings so their GP could talk to them on Zoom	• Not everybody can use technology • Resistance from older members to using laptops • Making appointments was difficult: ‘Press this for this, press that for that, press, press, press, press.’ • People avoided calling their GP, because they got fed up with what they had to go through to speak to them • Difficult to describe symptoms when you’re not seeing the GP • Members would come into the Clubhouse so a staff member could accompany them to online health appointments
• Ashiana Community Project (APC) never closed. They moved to an appointment system, and everything was done over the phone. ‘That’s where the access issues came in.’	• Elderly people couldn’t use the technology. They preferred face-to-face • Barriers of trust because some people were contacting APC for the first time • APC had many difficulties trying to help people sort out their benefits online that they couldn’t do themselves • Difficulty making GP appointments: ‘some clients couldn’t cope with the screening process. ‘There’s definitely discrimination in that process.’ • Receptionists are not sensitive enough with clients with complex needs. Some clients with mental health issues/problems communicating could not get an appointment • APC works with people who need access to their records. GP surgeries weren’t giving them appointments/medical summaries/documents, or charging excessively, £80 for a letter.’ Community Practice Nurses could not assist. • APC would give somebody a contact number, but they couldn’t get through, waited on the phone excessively for their GP or even 111 [NHS telephone number for immediate medical advice]

### Being treated unfairly and stigmatised when trying to get help

During the pandemic carers and service users were asked whether they felt they had ever been treated unfairly (e.g., to do with race/ethnicity/ country of origin or other reason), when trying to get help for their mental or physical health problems. Carers gave a mixed response, with a few stating they had not been treated unfairly, including one who thought that getting online care had contributed to their being treated more fairly than pre-pandemic in face-to-face appointments. Others said they were treated unfairly, during the pandemic, but this was not to do with race, but through feeling abandoned by their local mental health team. Another said her brother [SU65-site1], had ended up in a police station [described earlier, prior to the pandemic], and was eventually held in a secure unit for a considerable period. The family felt this was unfair, because he wasn’t given any help when he previously asked for it when his mental health started deteriorating.

Just under half of the service users raised explicit issues to do with racism and intersectional experiences of discrimination occurring in the wider society, which impacted their mental health negatively as well as unfairness in mental health care and treatment. These respondents talked about feeling they were treated differently to White people when they were ill because of their African, Caribbean, and Asian backgrounds. This difference in treatment involved difficulty accessing services and then subsequently finding themselves forcibly sectioned. Respondents described being subjected to force or abuse in their treatment, not being listened to, not being treated with respect, compassion and dignity, and with little understanding of their cultural background. An overarching theme was the way that past and/or ongoing experiences of racism, coupled with poor practice in mental health or healthcare generally, or with other institutions in society, were leading not only to mental health traumas, but to a deep distrust in wider systems and organisations and a sense of powerlessness.

Representatives from Mosaic and Ashiana talked about racism and intersectional forms of structural and interpersonal discrimination more broadly in society impacting people of African, Caribbean, and Asian backgrounds in different ways.

Ashiana said this discrimination is compounded for people with mental health conditions, who were “essentially invisible”, leading to them having a lower chance of securing financial support from statutory welfare benefits:

*“you’ve got people that have got PTSD, anxiety, depression – we have a lot of people on those kinds of benefits, and we found out a lot of them are being discriminated against because it was a hidden illness; it’s something you can’t physically always see unless you know what you’re looking for. So, when somebody would go for a benefit assessment they would always get rejected for the benefit…..[being told]…..‘You look fine to me’ ”.*

Mosaic said:

*“The education system disadvantages Black people, which leads to poorer Black communities which then in turn leads to lack of exercise and unhealthy diets (because you can’t afford it) and poorer overall health, which puts people at higher risk of COVID”.*

Service users gave several examples of how they experienced and viewed racism in their care:

Respondent SU4-site3 said he had never been *‘given any access to talking therapies’, whilst he had experienced several bereavements. ‘All these years of me being in here, I have never had alternative treatment. That is because I am Black. You can sugar-coat it like whatever, but that is the fact’*.

Respondent [SU6-site3] said they felt frightened of raising issues of racism, for fear of being told they were *‘playing the race card’.* When asked to clarify if they thought there was still unequal treatment in using services for minority groups or poor people, she said: *‘Yes. Personally, I would say 100% yes, but the thing is how do you prove it’.*

Other service users talked about experiencing aggression and abuse when using services because of racism (see experience of SU13-site3 mentioned above under Do health professionals know how to support you?).

Another service user said:

*‘My main concern would be that it’s a shame that, when you go and use these services, they don’t listen to what you’re saying, especially when you’re a Black man. If you go there and raise your voice or talk in a certain way, they say that you’re being aggressive, and that’s what they use against you’* [SU6-site2].

The negative experiences and microaggressions contributing to unfairness and discrimination in respondent’s treatment were often seen as being caused by staff who did not understand Black and Asian people, or communities who might be living in disadvantaged areas. The community worker from Mosaic Clubhouse noted:

*“White staff are often unrelatable for Black patients. Black people want to be treated by people who they can identify with, which increases trust”.*

Service users also made similar points about this issue:

*‘Most of the people that we talk to, as Black people, like I’m comfortable talking to you, but most of these people are, I’m gonna say White, middle class and they don’t know what we call inner city living’* [SU6-site2].

*‘People that I find could provide a bit of racism are unexperienced, young GPs in their 30s, or in their late 20s. They haven’t got experience working in poor areas, because the health issues are going to be wide and varied in poor areas’* [SU14-site3].

Being able to understand people’s cultural reference points were clearly seen as important:

‘*You have to do something about cultural training here if you are going to be a doctor from a different culture dealing with Black people in this particular postcode, because you need to be culturally aware’* [SU4-site3].

*‘It’s not just an ethnicity thing, but important for someone to be able to relate to you as you are with your background, as a Black man, regardless of if they are White or Black; they need to be able to relate to you in terms of who you are and your cultural reference points. Those things are clearly important’* [SU5-site3].

Mosaic reinforced the point that*:* “*Issues with communication between White and Black staff and different ways of communicating lead to misunderstandings*”. When providing care, Ashiana pointed out that staff need to be trained in cultural sensitivities, including people from different minority ethnic communities, who he thought also discriminate against each other and need to be trained just as much as the White population. Language barriers also need to be improved, through working with interpreters.

It was clear that wider intersectional experiences beyond race and ethnicity, such as socio-economic status, gender, having a care background or having a particular diagnosis, could lead to further stigma and discrimination. The participant below made this point very clearly:

*‘I feel that it’s not just race, but it’s tied in with other things. I think a lot is to do with your background. They have these inherent, ingrained racial prejudices, I think without them even realising it. I mean, it’s like, when they fill in forms for you, I notice all the time they put me on there as White. They’ve never bothered to ask me. One of the assessments was, I said I weren’t getting out – this is going back a few years where I’ve seen someone face to face and it said: ‘She appeared with a suntan but claimed she doesn’t go out.’ And I thought, ‘Suntan? That’s my natural skin colour.’. So, it’s that and its class, they’re just seeing you as working class from the north and they see you as lazy, fat, feckless, idol, uneducated, making things up, malingerers, health-seeking patient. They have this way of treating people, and especially women like me, with care backgrounds, who’ve had experiences of things like abuse, and they put horrible labels on them, without even their knowledge’* [SU1-site2].

### Involvement in improving race equality or health services

Given their health and care experiences, service users and carers were asked if they had ever been invited to be involved in helping to address race equality or improve healthcare services, and whether they would be interested in being involved. The majority of carers and service users said they had never been invited but would like to be involved.

Carers said:

*‘Yes, I am very passionate about equality, diversity, and inclusion, providing equality-sensitive services, dealing with these sides of things. So, I have taken part in some things, and I would like to take part in the future in as many things as possible to influence change’* [C1-site1].

*‘It is really not good that some people have been discriminated because of their colour or their race. Everybody should have the same rights to healthcare, to accessing the services. So, I think I would really love an opportunity to be able to do that’* [C1-site3].

Service users said:

*‘I would like to be involved in helping to improve things, but I do not want to be tokenistic. I have been in so many NHS meetings and events, so many times they take advantage of us ethnic minorities because it is like, ‘Oh yeah, let’s get an ethnic minority to sit and talk on the panel. Let’s get an ethnic minority to do a speech,’ or, ‘Let’s get an ethnic minority on our advisory board.’ It is like you just want to show people, ‘We’ve got an ethnic minority’. We know what we’re talking about. We’ve got a specialist. We’ve got somebody with lived experience. Let’s just tick a box,’ or, ‘We’ve got a peer researcher that’s black and brown and Chinese and disabled,’ and all the rest of it. It is like sometimes it is just showing and telling: We’re going to tell our funders that we’ve got BAME people, we’ve got disabled people, we’ve got refugees, we’ve got migrants.’ I just get sick & tired of it. I understand why, but I just feel like the way they go about it is not appropriate’* [SU13-site3].

## Discussion

Our research study found that more than two thirds of service users, and most carers, felt negatively impacted with their mental health during the pandemic, many very seriously. This situation was compounded by many participants feeling that they had little or no support, or finding much needed health and care services seriously disrupted or unavailable. Having face-to-face contact and access to their GPs was very important for participants. Good relationships with health professionals were seen to be built on trust, as well as being treated with compassion, dignity, and respect, and being listened to and not discriminated against. Not all participants felt they were understood and supported by GPs and professionals. Some felt their privacy and dignity had been impacted and/or that they had been treated rudely and aggressively. Half of participants described their care as unsafe in various ways and many participants raised explicit issues to do with racism and intersectional experiences of discrimination in using mental and physical health services. Many struggled with receiving care online or through telephone services, although some people described good experiences receiving their care in this way. Many participants had little or no support from family or friends prior to the pandemic and relied heavily on support services. Community organisations were described as playing an invaluable support role to many.

Not everyone felt negatively affected during the pandemic. Some service users reported that their care had not changed significantly and showed strong resilience with coping mechanisms. They were used to being on their own or able to change their behaviour positively and enjoyed staying indoors or keeping in contact with people on the phone or online.

People from minority ethnic backgrounds are more likely to be diagnosed with a SMI, which is closely associated with health inequalities. Health inequalities constitute avoidable and unfair differences in health status and determinants between groups of people due to demographic, socioeconomic, geographical, and other factors [13]. It is therefore crucial that people from racialised backgrounds who already experience considerable inequities, do not experience further inequities, or healthcare harm, in using services for their mental and physical health conditions that have been disrupted because of the pandemic, or because of racism or other intersectional discriminatory experiences.

A key challenge is how to address the considerable unjust health gap for racialised groups with SMIs in using primary and acute health services, as well as services in mental health trusts. During the spring 2020 peak of the COVID-19 pandemic in the UK evidence shows there was a marked reduction in help-seeking in primary care-recorded mental illness and self-harm, indicating untreated mental illness [17]. Many participants in our study reported the opposite, in highlighting significant issues in help-seeking in trying to access physical and mental health care across multiple systems which had been severely disrupted or were no longer available, with longer periods between appointments, shorter sessions, and poorer quality online support. This situation further impacted their mental and physical health. An important recent study [33] has shown that people who experienced disrupted access to healthcare (including appointments and procedures) during the COVID-19 pandemic were more likely to have potentially preventable hospital admissions. This evidence highlights the potential negative adverse implications for people with SMIs whose care was affected by the pandemic.

Our research participants cared deeply about their health and mental wellbeing and, wanting to stay well, and placed a high regard on getting the services and support they needed to enable them to do this. Engagement with vaccines from most participants showed that they strongly wanted to protect their health, which contrasted with reports of vaccine hesitancy in Black and some Asian communities during the pandemic [34]. This shows there is a real need to provide anti-racist and non-discriminatory culturally sensitive trauma-informed care for Black and Asian people with SMIs across the whole health and care system, in ways that are caring, compassionate and non-judgemental.

Study participants placed a strong emphasis on being able to have face-to-face access to GPs and services. Evidence shows Black, and minority ethnic communities are less likely to access mental health support in primary care (i.e., through their GP), more likely to end up in crisis care and up to 40 percent more likely to access mental health services via the criminal justice system than White people [35]. More generally, people with a physical health and mental health condition have been found to be up to four times more likely to attend an emergency department than the general population[36].

Analysis of recent General Practitioner Patient Survey (GPPS) data confirms a significant negative association between patient satisfaction and each of the main ethnic minority groups in the UK, which has persisted in recent years, but has not been shown to have changed during the pandemic [37]. Key supply/service-related factors found to have the most significant impact that account for observed ethnic differences in patient satisfaction at GP level include: ease of using GP website; being able to see preferred GP; being treated with care and concern; confidence of managing health condition; trust in health care professionals; and being involved in decisions about treatment. To access the GP website, patients would need access to relevant devices such as laptops, smart phones, and tablets. This was seen as being a problem for a disproportionately large proportion of patients belonging to minority ethnic groups who might lack financial resources to enable them to afford such devices. With regards to the issue of not being treated with care and concern, the authors suggest this is likely to be a result of misunderstandings or miscommunication between minority ethnic patients and their GPs. Therefore, the NHS could work towards implementing or reviewing the cultural competency component in the training of health care professionals, to help mitigate differences of cultural norms in the context of medical consultations. Allowing more time for GP consultation might also help GPs better understand any concerns.

Many of our study participants experienced several problems in trying to access and use online services because they did not have the skills, knowledge, ability, confidence, and finances to properly manage these digital and telephone management processes [highlighted in Table 3]. Despite our interviews being conducted online, some of our participants were not able to join Microsoft Teams or by phone unassisted and required help to do this either from family members or from Mosaic. This raises questions about people being able to participate online on their own, or in paying for Wi-Fi or phone costs themselves, or who do not own computer equipment. These experiences by Black and Asian participants in our study reflect wider evidence collected during the pandemic which showed that whilst many mental healthcare users were able to continue accessing some support by phone and video call (remote care), for others the shift to remote care presented significant barriers [38].

Our participants’ experiences suggest the need for greater targeted interventions by professionals to ensure that people with SMIs can access the health and care services they need to ensure their existing health conditions do not deteriorate further. There is a need for a system-wide approach to breaking down siloed (primary or secondary care) ways of working within local systems. This has been identified as a key priority in addressing health inequalities [39, 40]. The failure to provide both physical and mental healthcare in a timely and integrated manner for patients with mixed long-term conditions often leads to multiple crisis admissions and worsening physical and mental health which in turn re-impacts on service capacity.

The benefits of integrating care across boundaries (e.g., health, social care, employment, and housing) are understood [41]. But a National Confidential Enquiry Report has found good integrated care for people with mental health conditions often appears to remain the exception rather than the rule, with physical and mental healthcare systems largely disconnected. There is evidence that in many cases where mental health patients are being treated for physical disorders in a general hospital, they are seriously disadvantaged. The report exposes that in many cases the physical illness cannot be treated effectively until the mental illness is recognised and appropriately managed and many health problems were missed in the cases they looked at.

The authors of the report argue that patients who present with known co-existing mental health conditions should have them documented and assessed along with any other clinical conditions that have brought them to hospital. National guidelines should be developed outlining the expectations of general hospital staff in the management of mental health conditions. A key imperative is seen as the need to find ways of bridging the gaps between physical and mental health services if optimal care is to be delivered. Central to this is the need to have a workforce that is educated to understand these gaps and who are trained and supported to have the competence and the confidence to recognise and bridge the gaps at every level of the service to make a sustainable difference.

Patients have often been stigmatised with the term ‘revolving door’ when it is the healthcare system itself that can prevent timely access, or prematurely discharge patients, only for them to return later with significantly increased care needs [42]. The failure to listen to some participants in the study could also have been linked to 'diagnostic overshadowing', where there is a misattribution of physical symptoms to mental illness in the diagnostic process of people with mental illness [43].

Study participants wanted to see more joined up and integrated care plans across health and associated services (highlighted in the evidence under Impact of the pandemic on mental health and care services’ and in Table 4, Appendix 1, on Actionable recommendations from service users, carers, and community organisations), so clinicians can understand the whole picture of their mental and physical health. Evidence shows a positive relationship between continuity of care in mental health services and health outcomes among people with SMI [44]. Continuity of care with their GP and having a care plan are especially important in reducing hospital admissions [45] and addressing physical health needs [46].

With our participant data, this showed that many aspects of their care were impacted by the disjointed manner of accessing their appointments across mental health, secondary health services and social care. Individuals struggled to keep track of appointments and to travel to different appointments, particularly with multiple conditions and when they had a disability. In Table 4 (Appendix 1) service users, carers and the community organisations highlighted issues that were highly relevant for achieving better joined up care, such as the need for better signposting for help across the system, proper handover processes and procedures from one part of the system to another, to ensure people did not get lost, which was even more important for those who did not speak English. In addition to these areas, a community organisation participant felt strongly there needed to be a levelling up across the whole system in addressing the stigma associated with mental health and to address issues of racism and other forms of discrimination in an integrated way, as part of patient centred care, as everybody’s responsibility, rather than a side issue for some people.

An integrated and whole system approach is particularly important given that minority ethnic groups have been found to be more vulnerable generally to a higher rate of patient safety events in hospital and community settings compared to the mainstream population [47]. Psychiatric disease, including all psychiatric diagnoses, regardless of severity, have also been found to nearly double the risk of being a reported case of preventable harm [48]. Many of the study participants raised issues about the safety of their care across different services, ranging from not being able to access services, to receiving poor quality or discriminatory services and adverse treatment. A review by the National Confidential Enquiry into Patient Outcome and Death [41, 49] into the quality of physical healthcare provided to adult patients admitted to a mental health inpatient setting found room for improvement in the quality of care provided in 73% of cases as well as the systems that support and underpin the provision of care. As minority ethnic groups and particularly Black people are more likely to be over-represented in the mental health system, this is an important area to review, in improving the quality of their treatment.

Our study addresses an important gap in the literature in patient safety by showing how poor and discriminatory experiences impacting Black and Asian people with SMIs contribute to healthcare harm.

Experiences of racism and being treated unfairly were a strong focus of the study. These types of experiences are linked to an increased likelihood of developing depression; hallucinations and delusions; and if physical assault is involved, post-traumatic stress [50]. According to official NHS data the rate of people from Black backgrounds being restrained in mental healthcare has more than doubled in the past six years, widening the gap with other racially minoritised groups [51]. During the pandemic, with the use of police emergency powers, Black people were also three times more likely than White People to receive COVID-19 fines in England and Wales [52]. INQUEST who provide advice on investigations following a death in state detention or care in England and Wales, has found there is a clear need to ensure police scrutiny when it comes to the detention of minority ethnic groups [53].

Use of Mental Health Act 135 and 136 sections should be closely monitored given that there is no organisation that specifically regulates the police response to mental health detentions, and multi-agency reviews should be happening where there is repeated inappropriate use. Police training organisations, medical and nursing schools, and Approved Mental Health Professional (AMHP) training sites could work with those implementing the PCREF to help ensure that all patients are treated with dignity and respect and that all services are patient-centred and client-specific.

The PCREF provides a way for Mental Health Trusts in England to address racial disparities in care, in partnership with patients, carers and racialised communities. However, the PCREF only covers mental health trusts, when the issues raised by this study show that an integrated whole systems approach is needed across health and social care to support people with SMIs. In our interviews participants highlighted several areas where they thought action was important (see Appendix 1) and wanted to be involved in driving these changes. Their experiential knowledge can help to improve the safety and quality of health services. However, a key challenge will be to overcome historical barriers to how racialised groups are involved in coproducing solutions to race inequities across the whole health landscape, not just in mental health trusts. The benefits of Patient and Public Involvement (PPI) have long been seen as important in improving healthcare performance and quality [54, 55], however many groups have been underserved and underrepresented in these processes [56–58]. People from racialised backgrounds writing about the damaging effects of racism in mental health [25, 26] have highlighted that those facing discrimination and disadvantage have often faced additional obstacles in involvement processes [26]. Carers and service users in the study clearly valued the support and trusting relationships they were able to build with support groups, and voluntary and community organisations in the pandemic. These organisations can play a role in addressing race inequities alongside service users and carers across different health services, helping to produce new models of coproduction, including in governance and board structures in mental health Trusts so there is accountability in the delivery of services.

Gaining a better understanding of these systemic inequities, viewing them through an intersectional lens and linking them to patient safety improvement, would allow healthcare to aim for a more robust, inclusive, and realistic approach to healthcare quality and safety improvement and to recognise the importance of addressing these issues with racialised groups who tend to be excluded or under-represented in improvement processes [58].

## Strengths and limitations

This study was co-produced by a group of experts by experience and occupation. We were able to successfully interview service user and carer participants, and representatives from two community organisations, across three geographical areas who were purposively sampled by age (18 +), self-ascribed ethnicity (Black African, Black Caribbean, Bangladeshi, Indian or Pakistani descent), with a range of self-reported mental health diagnoses (depression, schizophrenia, bipolar disorder, or other psychotic disorder) and physical health long-term conditions. Black and Asian study participants are under-represented in mental health research, so this study gives a good indication of their experiences of using mental health services during a challenging phase of the COVID-19 pandemic in Britain. We captured these experiences broadly in equal numbers across participants from African, Caribbean, and South Asian backgrounds. A particular advantage of working in partnership with Mosaic was that we traversed the digital divide for people with SMI either unable to use or access digital technologies- so we heard from people who might otherwise not have been able to take part in a study like this.

Despite our broad inclusion criteria, we interviewed more participants from South London 17 than we did from Birmingham and Solihull and Manchester and fewer carers than we had intended. We also interviewed two rather than three community organisations covering all three geographical areas. Due to time constraints, we did not interview any healthcare providers. Participants were recruited through various means [by placing information on Trust websites, through community networks and social media]. We will not have captured data from people not accessing these channels.

## Conclusion

These findings demonstrate multiple points and processes along health and care pathways that have the potential to stack up and exacerbate the already considerable unjust health gap in mortality that exists for Black and Asian people with SMI, who often have multiple long-term physical health conditions. Reported experiences during the COVID-19 pandemic highlighted additional challenges for Black and Asian groups and potentially exacerbated pre-existing inequalities. This health gap was reflected in a marked increase in deaths during the pandemic. Persistent ethnic inequalities in access to mental healthcare, including higher levels of coercive pathways into care (via police or criminal justice routes), impacting Black African and Black Caribbean people, have been reported for decades. These types of negative experiences are further reflected in our study findings and highlight the risk of widening the health gap for these groups if greater action isn’t taken to mitigate systemic harms in several areas. Issues such as timely access to care (face to face and remote) and especially to GPs, being treated in a culturally appropriate manner with empathy, dignity and respect and being able to use services without experiencing coercive and undue force, racism or other forms of intersectional discrimination were important themes arising from this study. These poor experiences of using services, create systemic and enduring healthcare harms for racialised groups with SMIs and physical health conditions, that need to be addressed in a joined up and integrated way, not only in mental health trusts but across the health and care landscape and in safety and quality improvement processes, where issues of discrimination and how they impact different demographics are often not looked at and addressed, especially in partnerships with service users, carers, and communities from racialised backgrounds. Failure to address these issues of trauma and mental and physical ill health for Black and Asian groups will not only have a devastating impact upon these communities but is likely to exact much wider negative economic and social costs for the whole of society if not addressed.

## Supplementary Information

Additional file 1: Appendix 1 – Table 4 – Actionable recommendations by service users, carers, and community organisations.

Additional file 2: Appendix 2 – Service User Topic Guide.

Additional file 3: Appendix 3 – Carer Topic Guide.

Additional file 4: Appendix 4 – COREQ Checklist.

Additional file 5: Appendix 5 – GRIPP2 short form.

## Data Availability

The datasets generated and analysed during the current study are not publicly available to protect participants’ confidentiality. Although the interview transcripts have been pseudonymised, participants’ individual circumstances might be recognizable. Data are available from the corresponding author on reasonable request.

## Footnotes

1 The Mental Health Act 1983 (MHA) sets out criteria for detaining and compulsorily treating patients* with a mental disorder in hospital without their consent in England and Wales. (‘Mental Health Act 1983. Statute Law Database’.

2 See King’s Speech Programme 2024 [https://www.gov.uk/government/speeches/the-kings-speech-2024].

## Data availability

No datasets were generated or analysed during the current study.
