# Supplementary material for: Living less safely through the pandemic in England for people with serious mental and physical health conditions: qualitative interviews with service users and carers of Black African, Caribbean, and South-Asian descent
Source: BMC Public Health. 2024 Oct 5;24:2718. doi: 10.1186/s12889-024-20107-6 (PMC11452990; doi:10.1186/s12889-024-20107-6)
Supplement: Supplementary file 1 — Additional file 1: Appendix 1 – Table 4 – Actionable recommendations by service users, carers, and community organisations. [file 12889_2024_20107_MOESM1_ESM.docx]

**Appendix 1**

**Table 4 – Actionable recommendations by service users, carers, and community organisations.**

| **Actionable recommendations suggested by service users:** |
| --- |
| **Services** |
| More funding and GPs. Improved access to GPs, hospital appointments, social workers, and support services. Organisational shift within mental health services adopting a culturally sensitive approach |
| Thorough checks in A&E so people are not discharged prematurely and must return. Improved signposting for all health needs. |
| Protection from medical negligence. Senior staff should be held to account for racism and discrimination within trusts. |
| Holistic joined-up care model incorporating physical and mental health, trauma-informed care and wellness and designated holistic care-coordinators responsible for ensuring that all care needs are met. Accessible language to be used by all those providing care. |
| Technological systems within health to be more user-friendly, easier to input and access data. Face-to-face care is important, but online help is necessary out of hours and quicker. |
| Proper handover processes. When one professional leaves, they say goodbye and name who the next person will be. Transparency develops trust. |
| Heath services to be more transparent to develop greater trust and to hold public meetings that are open to other voices. Trusts to improve staff morale and model better interpersonal treatment which should impact on how staff treat patients. Transparency around decisions to use DNRs. |
| Training for police around race, mental health, and intersectionality. Police don’t always see people when they are well. Training could be done by community organisations such as Mosaic Clubhouse. |
| **Cultural sensitivity and extra support** |
| Help for people who do not speak English, who can’t access transport, and who have no support from family/ friends. Help with accessing care and appointments. Help with admin, technology and form-filling from a consistent service/person within the local authority. |
| Exemptions for people with severe/enduring mental health difficulties to be able to break isolation periods in a pandemic. |
| An overhaul of health funding policies and practice. Education about how to work with ethnic minorities and around racism/discrimination. Addressing staff attitudes and behaviours and developing increased cultural awareness. Assessment of health trusts for racial inequities by independent body. |
| Culturally appropriate advocacy to help get support for mental health needs when doctors “are just referring you round in circles” for support that is never actioned or provided. This needs to cover health, care, and social work. |

| **Actions suggested by or involving carers:** |
| --- |
| More education generally around mental health, and information on specific services and support. |
| Being kept up to date as a carer and more contact with staff and involvement in CPN meetings. |
| Being listened to as a carer and treated with respect, empathy, and compassion. Own needs considered. |

| **Actions suggested by community organisations:** |
| --- |
| **Stigma and mental health** |
| A need for a levelling up across the whole system in addressing the stigma associated with mental health and to address issues of racism and other forms of discrimination in an integrated way, as part of patient centred care, as everybody’s responsibility, rather than a side issue for some people.  ‘*Within the BAME communities, there’s still a lot of work that needs to be done around mental health and breaking down stigma and barriers around mental health and help seeking’ [community project 1].*  *‘I think the whole approach to mental health needs to change. It’s about levelling up on a national scale, so that there is respect for people with mental health [conditions sic], full stop. Addressing race equality then needs to be integrated into this wider approach’ [community project 1].* |
| **1. Culture and communities** |
| Provide culturally sensitive training for health care staff (mental and physical health care) that includes all ethnic groups, and with clear strategies for addressing intersectional experiences and language barriers and working with interpreters.  *‘There’s a need to address cultural awareness but there’s also kind of prejudice: how people are viewed and seen and stereotyped’ [community project 2].*  *‘A service user needs to be treated as an equal, not as inferior, not as below you. People who come to you with language barriers, IT barriers, health barriers. You should not look down upon them as, ‘Oh, he’s here again.’ No, that’s not it. Your job is to help them. Your job is not to judge them’ [community project 1].* |
| Adjust care provision based on cultural and personal needs of an individual rather than norms.  *‘There’s a need for a culturally sensitive and humane approach …. It’s having a more professional and humane approach, which is sensitive to each individual’ [community project 1].* |
| Racism and lack of trust |
| There are different reasons for people from racialised groups to have distrust in using services. Some examples given [by community organisation 2] were to do with:   - Racism and discrimination when using services   *‘Some are. Some are saying not to. So, there’s that. But also it’s that trust, isn’t there? I mean, it’s not wanting to be controlled and, if it is a chip, are you controlling me? [the idea that a chip could be inserted in people connected to the vaccine, was circulating as part of vaccine misinformation]. So, there’s all sorts of things, and I think that’s the lack of trust. There’s institutional racism, whether people want to accept it or not.  Black people are not understood’* [community organisation 2]*.*   - Tackling wider health disparities   *‘Black people are poorer because it’s not easy to get a job for some people. People of colour, it could be*  *education. It’s various reasons. I think exercise as well. So, if you’ve got no money, you’re not gonna*  *really be able to go and do exercise. You’ve not got no money, you’re not gonna really be able to eat*  *properly, so you’ll eat whatever. And it’s so expensive to live in London. If you’re living in London, you’ve*  *got to have a reasonably good job.   So, there’s loads of issues, and the lack of understanding. I mean, I*  *interview staff, and interviewing a white staff and interviewing a Black staff, there is a difference of the*  *way people communicate. But, if you’re used to a sort of way of people communicating, you’re gonna be*  *drawn to the person that you can relate to. So, when I’m interviewing, if there’s a Black person, I would*  *probably know more what they’re saying, ‘cos I can relate to that’* [community organisation 2]*.*  *‘People communicate with people that they can identify with. So, they would come to me and I would try and break that barrier’*[community organisation 2]*.*   - Forcible treatment and not being treated with kindness, empathy and respect. An example given was that one person would not take the vaccine after being forcibly injected previously as part of his mental health care.   *‘I think, as well, in mental health, when you’re ill, some of the guys here have had to be forcefully given injections, and I suppose they’ve got a choice now.  So, they’re not gonna choose to’* [community organisation 2]*.*   - *Religious influence*   *‘People like to quote the Book of Revelations, and I’m like, ‘But you quote bits you want to’ [community organisation 2].* |
| **Financial support and partnership working with community organisations/public** |
| Fund and support community organisations who are equipped to work in culturally sensitive ways, that use staff, advocates and ambassadors who are from racialised and under-represented groups and thus reduce health inequalities and systemic racism. Research organisations should not keep coming back to community organisations with the same questions, but demonstrate that they are going beyond consultation and coproducing research with groups, and demonstrating how findings are being implemented.  *‘You need to get a lot of organisations involved, definitely, but BAME-led organisations, where BAME people sit at the top – as they have a grassroot connection, because, to be honest with you, there’s a lot of organisations that have BAME employees and have an element of BAME, but they’re still driven by different perceptions at the top’ [community project 1].* |
