# Supplementary material for: Living less safely through the pandemic in England for people with serious mental and physical health conditions: qualitative interviews with service users and carers of Black African, Caribbean, and South-Asian descent
Source: BMC Public Health. 2024 Oct 5;24:2718. doi: 10.1186/s12889-024-20107-6 (PMC11452990; doi:10.1186/s12889-024-20107-6)
Supplement: Supplementary file 3 — Additional file 3: Appendix 3 – Carer Topic Guide. [file 12889_2024_20107_MOESM3_ESM.doc]

**Appendix 3:**

**Ethnic inequalities in mortality and service use in people with mental disorders and multimorbidities during the COVID-19 outbreak**

**(COV-EIMM Study)**

## **Topic Guide for Carers**

| **Research aims to explore**   - the impact of COVID-19 on   - ethnic health inequalities   - service use and care pathways   - on mortality in adults - the mechanisms through which inequalities identified have been perpetuated - actionable recommendations to   - prevent further inequalities   - to improve patient safety |
| --- |

| **Pre-interview checklist**   - Confirm with the researcher who set up the interview any input you want from them (e.g. they could help with taking consent and answering questions about the study) - Confirm with the researcher recording how you will contact them if needed during interview - Confirm with the researcher organising the interview how you will check in with them after the interview   **Have a copy to hand of:**   - Participant Information Sheet - Consent Form - Topic Guide - Contact Card Resource that may be helpful to refer to if the person is distressed, including details for: Samaritans, CALM, MIND and Rethink   **Starting the interview: interviewer prompts:**   - Introduce self and person recording and explain role. - Check the participant is happy with the interview technology. If they are participating online, remind them they have the choice to have their video on or off. - Ask the participant if they have read the Participant Information Sheet, and if they have any questions. - Capacity to consent questions: ask about aspects of Participant Information Sheet (refer to slides and decide which questions you would like to ask). - (If this has not previously been discussed), confirm with the participant that the researcher will start recording before taking formal consent - Complete the Consent Form by reading out each statement (the person recording will screen share the form) and asking person to verbally agree after each statement. - Stop recording and explain that it will be saved. The interview will be recorded separately and that recording will be sent for transcription. When we have checked that the interview transcription is accurate and we have pseudonymised any names, we will destroy the recording of the interview. |
| --- |

**Introduction**

Thank you so much for agreeing to be interviewed. As you know the purpose of the interview is to explore the experiences of service users from Black African, Black Caribbean, Indian, Pakistani and Bangladeshi descent living with multiple long-term conditions and mental health problems, as well as carer and healthcare provider perspectives, from three geographical places in England (Birmingham and Solihull, Manchester and South London). The study is being carried out in order to understand the reasons underlying ethnic inequalities in mortality and service use during the Covid-19 pandemic. This will include looking at access and barriers to care and discrimination within health services. Our aim is that recommendations resulting from the research will help to improve health care and make it safer.

There are no right or wrong answers, as everyone’s experience will be different. What you say will be kept completely confidential and we’ll anonymise the information you give us, so we won’t use your name or say anything that could identify you, especially in anything we write. Please be as open as you feel comfortable, and if you want to skip a question or take a break at any point, please let me know.

Firstly, I’d like to ask you for some information about yourself, in order to help us understand better the context of your answers to the interview questions.

**START RECORDING**

### **Background**

1. Who do you currently live with?

- the person I am supporting/caring for
- partner
- children
- other family
- friends?

1. Please indicate the nature of your main caring responsibilities /what relationship you have?

- Child or minor dependant
- Partner – marriage / civil
- Partner - other
- Parent
- Sibling / brother or sister
- Other (please specify) ___________________________________________________

1. Are you currently supporting someone/or were you supporting someone using mental health or other health services Y/N?
2. Can you describe the services you have been supporting them with?
3. When was your last contact with these services in your support/caring role?
4. Have you been able to get support in your caring role from any other place outside of the NHS [e.g. voluntary/community sector organisations, family, friends, carer, other]?
5. Has this changed since the COVID-19 Outbreak [e.g. since January 2020]?
6. Which region do you live in?

- London?
- Birmingham?
- Manchester?

1. Do you consider yourself vulnerable to COVID-19 because of any underlying health conditions which has impacted your carer role?

*Prompt:*

- Can you tell me more about this?
- which health conditions
- any changes made as a result of being vulnerable

1. Have you been isolating as a result of the COVID-19 outbreak and has this impacted your carer role?

*Prompts:*

- Have you been officially advised to shield?
- If so, have you been affected by any changes to shielding guidelines over time?
- Was it your own decision to isolate and why?
- Do you have any specific concerns around shielding or self-isolating (e.g. being forced to work or have face to face contacts or issues with financial support)?
- Have you received any additional support whilst isolating?
- Has isolation affected your health, mental or physical?

1. Have you been limiting your social contact and if so in what way?

*Prompts:*

- With family or friends?
- What social contacts have you had?
- Has how this affected you and the person you have been caring for/supporting?
- What could have helped make this better?

1. Have you or the person you have been caring for/supporting had any COVID-19 symptoms or been diagnosed with the virus?

Symptoms include:

- continuous cough (coughing a lot for more than an hour, or three or more coughing episodes in 24 hours)
- High temperature
- Shortness of breath or trouble breathing
- Fatigue
- Loss of sense of smell or taste
- Diarrhoea
- Abdominal pain
- Loss of appetite

### **Interview Questions**

1. Can you tell me a bit about the mental and physical health conditions of the person you have been supporting/caring for?

*Prompts:*

- Have you faced additional challenges in supporting them during the COVID-19 outbreak?
- Have their mental and physical health conditions been affected during the outbreak?

1. Has the COVID-19 outbreak affected the care of the person you have been supporting/caring for and was this care different to the that provided previously?

*Prompts:*

- mental healthcare?
- primary care (GPs etc.)?
- physical healthcare (e.g. from hospital appointments, diabetes/ cardiac services etc or whatever people are receiving for their physical health conditions)?
- non-NHS care/ input (through day centres, social care/ social services, carers support, personalised support etc.)

1. Have you experienced any problems during the outbreak trying to get healthcare and/or other support for the person you are caring for?

*Prompts:*

- What kind of support, from whom?
- Access to transport etc?
- Explore whether any care has been withdrawn completely
- what care is no longer available?
- what is being provided face to face, by telephone or online.
- Have they tried or wanted to access services during pandemic and not been able to for any particular reason? (e.g. many people may have stayed away from services because they were justifiably worried about catching COVID + public health messages to tell people to stay away)

Has the person you have been supporting/caring for had to get their care by telephone or online during the COVID-19 outbreak and can you describe your experience of this?

*Prompts*:

- How this has worked?
- Have there been any problems?
- If needed, have you been able to get any training or help by care coordinators to access services or support?

Have you received any information or advice that has affected the way you use health services as a carer?

*Prompt* if so, where from:

- Official information
- Family or friends
- Social media (twitter / WhatsApp / Facebook / other)
- Places of worship / faith groups
- Usual support network
- Community networks

1. During the COVID-19 outbreak have healthcare professionals in general (GPs, psychiatrists, care co-ordinators) understood how to work with you as a carer in supporting someone with mental and physical health conditions/concerns?

*Prompts:*

- Do they take your concerns seriously?
- Thinking back to a time prior to the COVID-19 outbreak do you think this has changed?

During the COVID-19 outbreak, are there any specific things that have made accessing healthcare or support for the physical health condition of the person you are supporting/caring for more difficult for you?

Who would you go and see, if anyone, if you had any concerns about the physical health of the person you are supporting/caring for?

*Prompts:*

- Has this changed during the COVID-19 outbreak?
- If not about their GP or other health professional
- who else would you see?
- friends?
- family?
- religious or community leaders?
- pharmacist?
- Community Mental Health Team?

1. During the COVID-19 outbreak can you think of a time when you may have seen the health worker of the person you are supporting/caring for, about their physical health condition?

Ask which: GP/ care coordinator/ A&E/ other health professional/ keyworker/

specialist physical healthcare?

*Prompts:*

- Can you think of an example where this was dealt with well?
- Can you think of an example where this was not dealt with well?
- If not dealt with well, why do you think this was the case?
- How did this compare to your experiences prior to the COVID-19 Outbreak?

If talking about their GP also ask:

- How have you found it making an appointment?
- Are appointments long enough?
- Do you feel your GP has taken the concerns you were raising about the physical health of the person you are supporting/caring for seriously?

If you were not able to access your usual services in providing carer support, have you been able to get other types of health support? Are there things you think could have been done differently?

1. Have you been able to gain support from friends, family, or anyone else during the COVID-19 Outbreak in your role providing supporting/caring for someone with a mental health condition?

- Neighbours?
- Religious networks?
- Explore if they have become more socially isolated as a result of the COVID-19 outbreak and impact of this on their mental and physical health.

1. During the COVID-19 outbreak can you think of a time when you felt you or the person you were supporting/caring for were treated unfairly [e.g. to do with race/ethnicity/ country of origin or other reason], when you were trying to get help for their mental health or physical health problem?

*If participant responds ‘yes’ but is not specific, then prompt as follows:*

- What might you have been treated unfairly about and how?
- Have you had these types of experiences before the outbreak?
- Have things changed?

1. Do you feel that the person you are supporting/caring for has ever received unsafe care for their mental or physical health during the COVID-19 outbreak?

*Prompt:*

- If yes, please describe any specific examples of unsafe care.

1. What do you think about recent vaccine developments for COVID-19?

- Do you know if the person you are supporting/caring for would consider taking this?
- If not, do you know why?

1. Can you tell us whether the person you have been supporting/caring for has felt able to comply with mask wearing and other infection prevention and control measures (e.g. face coverings, space/social distancing, handwashing/sanitising etc)?

- If not, do you know why?

1. Given your experiences of using healthcare services during the COVID-19 outbreak as a person providing carer support, can you think of any actions that could be taken to improve your experiences or the person you are caring for?
2. Have you ever been invited to be involved in helping to address race equality or improve healthcare services and is this something you would be interested in being involved with as a carer?

*Prompt:*

- If not interested, is there any particular reason why?

**STOP RECORDING**

| **After recording has stopped …**   - Interviewer to thank the participant and hand over to the university researcher. - The university researcher will ask the questions on the demographic question form and note the information on a spreadsheet. - The university researcher will check whether the participant has any remaining questions about the research. - The university researcher will reassure the participant about confidentiality and anonymity. - The university researcher will ask if they would prefer a £20 bank transfer or shopping voucher. |
| --- |
