# Supplementary material for: Living less safely through the pandemic in England for people with serious mental and physical health conditions: qualitative interviews with service users and carers of Black African, Caribbean, and South-Asian descent
Source: BMC Public Health. 2024 Oct 5;24:2718. doi: 10.1186/s12889-024-20107-6 (PMC11452990; doi:10.1186/s12889-024-20107-6)
Supplement: Supplementary file 4 — Additional file 4: Appendix 4 – COREQ Checklist. [file 12889_2024_20107_MOESM4_ESM.docx]

Appendix 4:

Consolidated criteria for reporting qualitative studies (COREQ): 32-item checklist

Please indicate in which section each item has been reported in your manuscript. If you do not feel an item applies to your manuscript, please enter N/A.

For further information about the COREQ guidelines, please see Tong *et al.*, 2017: <https://doi.org/10.1093/intqhc/mzm042>

| **No.** | **Item** | **Description** | **Section #** |
| --- | --- | --- | --- |
| **Domain 1: Research team and reflexivity** | | | |
| Personal characteristics | | | |
| *1.* | Interviewer/facilitator | Which author/s conducted the interview or  focus group? | Josephine Ocloo (JO), Dina Choudhury (DC), Leroy McAnuff (LM), Sandra Jayacodi (SJ). (P8) |
| *2.* | Credentials | What were the researcher's credentials? *E.g.*  *PhD, MD* | PhD (JO), Mx (DC, LM, SJ). |
| *3.* | Occupation | What was their occupation at the time of the  study? | University Researcher (JO) |
| *4.* | Gender | Was the researcher male or female? | 3 Female, 1 Male (P8) |
| *5.* | Experience and  training | What experience or training did the researcher  have? | Professional experience (JO), Lived experience and tailored training before the interviews (DC, LM SJ). (P8) |
| Relationship with participants | | | |
| *6.* | Relationship  established | Was a relationship established prior to study  commencement? | No relationship was established prior to study commencement. |
| *7.* | Participant knowledge of the interviewer | What did the participants know about the researcher? *E.g. Personal goals, reasons for*  *doing the research* | Reasons for doing the research, dissemination plans. |
| *8.* | Interviewer characteristics | What characteristics were reported about the interviewer/facilitator? *E.g. Bias, assumptions,*  *reasons and interests in the research topic* | JO is a researcher and activist while DC, LM and SM are lived experience researchers with an interest in mental health. (P8) |
| **Domain 2: Study design** | | | |
| Theoretical framework | | | |
| *9.* | Methodological orientation and theory | What methodological orientation was stated to underpin the study? *E.g. grounded theory, discourse analysis, ethnography,*  *phenomenology, content analysis* | Participatory action research.  (P6,7,11) |
| Participant selection | | | |
| *10.* | Sampling | How were participants selected? *E.g. purposive,*  *convenience, consecutive, snowball* | Participants were purposively sampled by age (18+), self-ascribed ethnicity (Black African, Black Caribbean, Bangladeshi, Indian or Pakistani descent), self-reported mental health diagnoses (depression, schizophrenia, bipolar disorder, or other psychotic disorder) and physical health comorbidities. (P9) |
| *11.* | Method of approach | How were participants approached? *E.g. face-*  *to-face, telephone, mail, email* | Participants were approached via e-mail and telephone. (P9) |
| *12.* | Sample size | How many participants were in the study? | 30 participants were interviewed for this study (24 service users and 6 carers). (P12-13) |
| *13.* | Non-participation | How many people refused to participate or  dropped out? What were the reasons for this? | Some participants did not show up to their scheduled interviews without canceling or specifying a reason. |
| Setting | | | |
| *14.* | Setting of data  collection | Where was the data collected? *E.g. home, clinic,*  *workplace* | The interview took place online and were conducted from home. Most participants also took part from home, except for some who either struggled with the technology or didn’t have internet or a device and therefore joined the call from a community organization. (P10) |
| *15.* | Presence of non-  participants | Was anyone else present besides the  participants and researchers? | Hannah K Dasch (HD), a research assistant, was present during the interviews conducted by the lived experience researchers in order to assist with technology and consent as well as provide support as needed. (P10) |

| *16.* | Description of sample | What are the important characteristics of the  sample? *E.g. demographic data, date* | Anonymised data in Table 1 based upon participant location, ethnic background, gender, sexual orientation, age and disability. (P12-13) |
| --- | --- | --- | --- |
| Data collection | | | |
| *17.* | Interview guide | Were questions, prompts, guides provided by  the authors? Was it pilot tested? | Yes, the interview guide was semi-structured with various prompts available, and these were discussed with the lived experience researchers who conducted the interviews during the interview training prior to study commencement. The interview guide was pilot tested by the lived experience researchers on research assistant (HD). (P8,10) |
| *18.* | Repeat interviews | Were repeat interviews carried out? If yes, how  many? | No. |
| *19.* | Audio/visual recording | Did the research use audio or visual recording  to collect the data? | Both audio and visual recording were used during the interviews after consent had been given. (P10) |
| *20.* | Field notes | Were field notes made during and/or after the  interview or focus group? | No. |
| *21.* | Duration | What was the duration of the interviews or  focus group? | The duration of the interviews ranged from 30-90 minutes. (P10) |
| *22.* | Data saturation | Was data saturation discussed? | Yes. (P8) |
| *23.* | Transcripts returned | Were transcripts returned to participants for  comment and/or correction? | No. |
| **Domain 3: analysis and findings** | | | |
| Data analysis | | | |
| *24.* | Number of data  coders | How many data coders coded the data? | Peer researchers (DC, SJ, YT) fed into the coding framework and three university coders (JO, HD and RS) coded the data. (P11) |
| *25.* | Description of the  coding tree | Did authors provide a description of the coding  tree? | No.  JO coded all the transcripts in NVivo 12 using a coding frame of inductive themes identified at the meeting and subsequently when coding in NVivo and deductive codes based on questions in the topic guides. (P11) |
| *26.* | Derivation of themes | Were themes identified in advance or derived  from the data? | Themes were derived from the data. (P11) |
| *27.* | Software | What software, if applicable, was used to  manage the data? | NVivo 12. (P11) |
| *28.* | Participant checking | Did participants provide feedback on the  findings? | Lived experience researchers provided feedback on the findings and participants were invited to a dissemination event in which the findings were discussed. (P12) |
| Reporting | | | |
| *29.* | Quotations presented | Were participant quotations presented to illustrate the themes / findings? Was each  quotation identified? *E.g. Participant number* | Yes. Quotations were identified by adding e.g. service user/carer, location (South London, Birmingham & Solihull or Manchester) and the participant number. (P12-37) |
| *30.* | Data and findings  consistent | Was there consistency between the data  presented and the findings? | Yes. (12-37) |
| *31.* | Clarity of major  themes | Were major themes clearly presented in the  findings? | Yes. (P12-37) |
| *32.* | Clarity of minor  themes | Is there a description of diverse cases or  discussion of minor themes? | Yes. (12-37) |

When submitting your manuscript via the online submission form, please upload the completed checklist as a Figure/supplementary file.

If you would like this checklist to be included alongside your article, we ask that you upload the completed checklist to an online repository and include the guideline type, name of the repository, DOI and license in the *Data availability* section of your manuscript.

Developed from: Allison Tong, Peter Sainsbury, Jonathan Craig, Consolidated criteria for reporting qualitative research (COREQ): a 32-item checklist for interviews and focus groups, International Journal for Quality in Health Care, Volume 19, Issue 6, December 2007, Pages 349–357, <https://doi.org/10.1093/intqhc/mzm042>
