# Supplementary material for: Living less safely through the pandemic in England for people with serious mental and physical health conditions: qualitative interviews with service users and carers of Black African, Caribbean, and South-Asian descent
Source: BMC Public Health. 2024 Oct 5;24:2718. doi: 10.1186/s12889-024-20107-6 (PMC11452990; doi:10.1186/s12889-024-20107-6)
Supplement: Supplementary file 5 — Additional file 5: Appendix 5 – GRIPP2 short form. [file 12889_2024_20107_MOESM5_ESM.docx]

**Appendix 5:**

**Table 2** **GRIPP2 short form**

For further information about the COREQ guidelines, please see [Tong *et al.*, 2017](https://researchinvolvement.biomedcentral.com/articles/10.1186/s40900-017-0062-2): <https://doi.org/10.1093/intqhc/mzm042>

| Section and topic | Item | Reported on page No |
| --- | --- | --- |
| 1: Aim | **Report the aim of the study**   - To understand the reasons underlying ethnic inequalities in mortality and service use during the Covid-19 pandemic for service users and carers of Black African, Black Caribbean, Indian, Pakistani, and Bangladeshi backgrounds living with multiple serious long-term mental and physical health conditions. - To understand issues of access and barriers to care, and discrimination within health services for these groups. | Page 1 |
| 2: Methods | **Provide a clear description of the methods used for PPI in the study**  We drew upon a community peer research approach. This is a participatory method which enabled us to engage with the communities directly affected by the research and enable them to take part in designing, conducting, analysing, and disseminating the research. The research was co-produced by a group of experts-by-experience and occupation: three university researchers and four peer researchers with lived experience of using mental health services.  Peer researchers were involved in all aspects of the study, the initial design through discussions in the Steering Group, feeding into and reviewing the topic guides, helping with participant recruitment, conducting interviews and analysis, write-up for publication and involvement with an online dissemination event. Incentives for peer researcher participation included opportunities to engage with the work throughout all stages of the study and to be paid for this at a rate of £25 per hour, as well as participating in training sessions if they wished and being involved in wider dissemination of the findings.  Peer researchers DC, LMN and SJ were provided with qualitative research and interview training in an online meeting, after email communication with the peer researchers to see if they would find this useful. Peer researchers read a selection of different transcripts to discuss potential themes which fed into the coding framework.  Study participants were invited to an online dissemination meeting co-facilitated with our peer researchers to discuss the findings and make recommendations.  Peer researchers verbally commented on the manuscript, with the support of a university researcher who wrote up their comments and incorporated them into the paper. | Pages 6-12 |
| 3: Study results | Outcomes—Report the results of PPI in the study, including both positive and negative outcomes  Key positive outcomes from collaborating with peer researchers enabled experiential knowledge to shape the whole design of the study, in particular helping to get the focus of the questions right, being able to recruit racialised groups meeting the study criteria and to help identify key themes, conclusions, and outcomes for action. Targeted researcher support for the peer researchers was crucial to enabling their involvement, although this presented challenges in terms of the rapid timescales of the study. | Pages 6-12 |
| 4: Discussion and conclusions | Two peer researchers are authors on the paper and so had a strong influence on the whole paper and the discussion and conclusions |  |
| 5: Reflections/critical perspective | Comment critically on the study, reflecting on the things that went well and those that did not, so others can learn from this experience  As part of the participatory approach, the study team experts by experience and occupation wrote individual reflective logs to capture experiences of the research process. University researchers self-identified as White females and Black female. In terms of our collective standpoints, there was a strong recognition of the need to address imbalances of power by providing extra ongoing support for peer researchers in the research. For example, getting in touch by telephone and posting paper documents rather than just using email, creating opportunities to contribute verbally via individual online meetings rather than in writing, and affording sufficient time for preparation and debriefing. The involvement of the university researcher from a racialised background, was seen as a particular strength within the research team in enabling open discussions about emerging themes of racism in the interview data. Peer researchers fed back about their experiences of recruiting and interviewing, for example, recommending that the language be simplified in the consent statements which they had to read out to participants. Challenges for the university team included being able to provide extra time to allow the peer researchers to be involved, whilst getting the balance right in terms of communicating with peer researchers about work that needed to be progressed and completed to tight deadlines. | Pages 11-12 |

PPI=patient and public involvement
